# Development and Evaluation of ClientBot: Patient-Like Conversational Agent to Train Basic Counseling Skills

**DOI:** 10.2196/12529

**Published:** 2019-07-15

**Authors:** Michael J Tanana, Christina S Soma, Vivek Srikumar, David C Atkins, Zac E Imel

**Affiliations:** 1 Social Research Institute College of Social Work University of Utah Salt Lake City, UT United States; 2 College of Education University of Utah Salt Lake City, UT United States; 3 School of Computing University of Utah Salt Lake City, UT United States; 4 Psychiatry and Behavioral Science University of Washington Seattle, UT United States

**Keywords:** psychotherapy training, interactive learning, conversational agents, deep learning

## Abstract

**Background:**

Training therapists is both expensive and time-consuming. Degree–based training can require tens of thousands of dollars and hundreds of hours of expert instruction. Counseling skills practice often involves role-plays, standardized patients, or practice with real clients. Performance–based feedback is critical for skill development and expertise, but trainee therapists often receive minimal and subjective feedback, which is distal to their skill practice.

**Objective:**

In this study, we developed and evaluated a patient-like neural conversational agent, which provides real-time feedback to trainees via chat–based interaction.

**Methods:**

The text–based conversational agent was trained on an archive of 2354 psychotherapy transcripts and provided specific feedback on the use of basic interviewing and counseling skills (ie, open questions and reflections—summary statements of what a client has said). A total of 151 nontherapists were randomized to either (1) immediate feedback on their use of open questions and reflections during practice session with ClientBot or (2) initial education and encouragement on the skills.

**Results:**

Participants in the ClientBot condition used 91% (21.4/11.2) more reflections during practice with feedback (*P*<.001) and 76% (14.1/8) more reflections after feedback was removed (*P*<.001) relative to the control group. The treatment group used more open questions during training but not after feedback was removed, suggesting that certain skills may not improve with performance–based feedback. Finally, after feedback was removed, the ClientBot group used 31% (32.5/24.7) more listening skills overall (*P*<.001).

**Conclusions:**

This proof-of-concept study demonstrates that practice and feedback can improve trainee use of basic counseling skills.

## Introduction

### Mental Health Treatment in the United States

In 2014, 43 million adults (18% of the population) in the United States were diagnosed with a mental illness and 21 million Americans with a substance use disorder [1]. Despite a severe need for treatment, less than half of those individuals received mental health services [2]. There is a severe shortage of mental health providers [3], and thus, seeking care can involve many calls to providers [4] and wait times that are longer than the duration of many acute mental health episodes [5]. Moreover, training licensed master’s- or doctoral-level psychotherapists is an expensive and time-consuming process. There is incredible societal need to reduce the burden of mental illness and addiction, but a limited workforce and barriers to the rapid and effective training of providers create challenges for addressing these concerns.

### Psychotherapy Training

Psychotherapy training typically includes didactic classroom-based academic instruction, skills practice via role-plays with peers, viewing and discussing recordings of experienced psychotherapists, and clinical supervision, with supervision playing the most significant role [6,7]. Ideally, supervision includes review of recorded sessions and specific performance-based feedback from a competent supervisor. Gold-standard training for licensed therapists includes a workshop-based introduction to a treatment approach and then posttraining support, including coaching and performance-based feedback via a behavioral coding fidelity measure. There is strong evidence that providing ongoing performance-based feedback via behavioral coding to therapists results in skills acquisition and retention (eg, [8]). However, this process is slow and labor intensive (eg, in some cases 4 or 5 times the length of the session) [9]. Consequently, specific and objective feedback based on behavioral coding is rarely used in training.

Even when feedback is available, it usually occurs long after the actual performance of the therapy and is generally vague [10]. Supervision and training primarily rely on the therapist’s self-report of what occurred in client sessions [11]. Supervision can be general and highly selective in nature, as opposed to targeting specific behaviors [6,7]. The Beutler study [6] observed, “trainees are provided with suggestions for addressing crises and major problems too late to benefit the patient, and even then, the supervision is typically poorly focused and provides few means to assess improvement.” For example, training in basic interviewing/active listening sills (eg, open questions and reflections) is foundational to training in mental health counseling and much of the medical field generally [12,13], and Motivational Interviewing (MI), which is partly based on the use of these skills, is a widely used evidence-based treatment [14]. However, treatments such as MI typically rely on workshops where opportunities for practice and feedback are fairly limited.

Research from cognitive science suggests delayed, nonspecific feedback is not sufficient to promote learning and develop expertise [15]. It has long been established that immediate feedback on specific behaviors is an optimal part of a training regimen with large, positive effects on learning [16]. When this feedback is done correctly, it can outweigh other powerful effects on learning, such as cognitive ability and socioeconomic influences [15]. Typical psychotherapy training and supervision does not meet these optimal conditions, and trainee therapists rarely receive feedback as they are performing the skills themselves.

Another practical difficulty with training therapists is to provide initial skills practice without relying on actual clients. Standardized patients, who are actors that simulate clients and their problems [17], reduce the risk of harming clients with untrained therapists, but they can be expensive or difficult to train. Screening for low-severity clients is another alternative, though they can be difficult and time-consuming to recruit (requiring senior staff time to screen and supervise). Despite best efforts and screening, these clients may ultimately reveal severe mental health concerns. In summary, from the view of the cognitive science literature, ideal psychotherapy training would include many opportunities to practice, with immediate performance-based feedback. However, many practical barriers currently prevent psychotherapy training from meeting these conditions.

### Machine Learning and Psychotherapy

The field of computer science, and specifically machine learning, may provide potential solutions to availability of clients and lack of immediate feedback. Machine learning describes the process of creating algorithms through which a computer continues to learn from the algorithm without continued human interaction [18]. Recent developments in the field of machine learning and artificial intelligence may present solutions to standardizing and scaling up psychotherapy training [19]. Natural Language Processing (NLP) is a subgroup of machine learning, whereby the goal is to “learn, understand, and produce human language content” computationally [20], and recent work has begun to apply NLP to the training of mental health providers.

#### Natural Language Processing–Based Feedback

First, improvements in NLP have allowed computational models to replicate behavioral coding evaluations of psychotherapy that typically require trained human evaluators [21,22]. Currently, NLP models are able to identify key aspects of MI [23] (eg, questions and reflections)—an evidence-based psychotherapy for substance abuse problems—with similar accuracy to human raters [24]. This new technology allows for the possibility of a computer giving immediate feedback that would not be possible with human raters [25,26]. These new technologies create an opportunity to provide trainees with more rapid feedback that does not rely on resource-intensive human supervision.

#### Neural Conversational Agents as Standardized Patients

In addition to NLP-based evaluation of therapy, conversational agents may provide a computerized environment for practicing skills, potentially replacing standardized patients in some context. Conversational agents are computer programs that are intended to interact with a real person using language [27]. Early conversational agents relied on rule-based programming with long lists of if-then rules, which limits the ability to adapt a conversational model to a new domain. A recent, major innovation in computer-modeled conversational agents were algorithms that could generate plausible speech without relying on human-generated rules (ie, neural conversational models) that self-teach how to engage in dialogue, learning from a large corpus of examples (eg, recursive neural networks) [28]. Conversational agents have been utilized for training in the medical field [29] but have not yet been applied to training in psychotherapy.

Although conversational agents have not been used in psychotherapy training, there have been attempts to utilize technology to support skills practice and assessment. For example, the Rosengren study [30] created a system, whereby therapists were presented with standardized patient video vignettes and were asked to respond using MI skills (in written form). Their responses were later scored by human raters for MI fidelity [31]. This method has the advantage of providing a truly standardized patient; however, the patient did not respond to the therapist, preventing a more natural clinical exchange. In addition, the system requires a human to score the responses, delaying the receipt of feedback. The Baer study [32] developed a similar system, whereby therapists were presented with video clips and asked to respond as their therapist. Again, responses were scored later by a human for adherence to MI best practices. Thus, neither system has the ability to provide feedback immediately after each therapist response. New NLP models have created the opportunity for simulating a standardized patient without the cost of recruiting and training human patients.

### This Study

To address the challenges related to the need for scale and immediacy in training new skills in psychotherapy, we developed and evaluated a Web–based system that uses machine learning–based feedback for training 2 specific counseling skills: open questions and reflections. The feedback is embedded into a text–based neural conversational agent, developed to be a standardized patient. Thus, the skills training relied on an automated standardized patient—ClientBot—which provided real-time feedback to trainees on their utilization of specific counseling skills. We randomized nontherapist participants to receive real-time feedback on skill use (or not) and hypothesized that participants in the feedback condition would use more desirable counseling skills (ie, open questions and reflections) after training has ended than in the no feedback condition.

## Methods

### ClientBot Development and Overview

**Figure 1:**
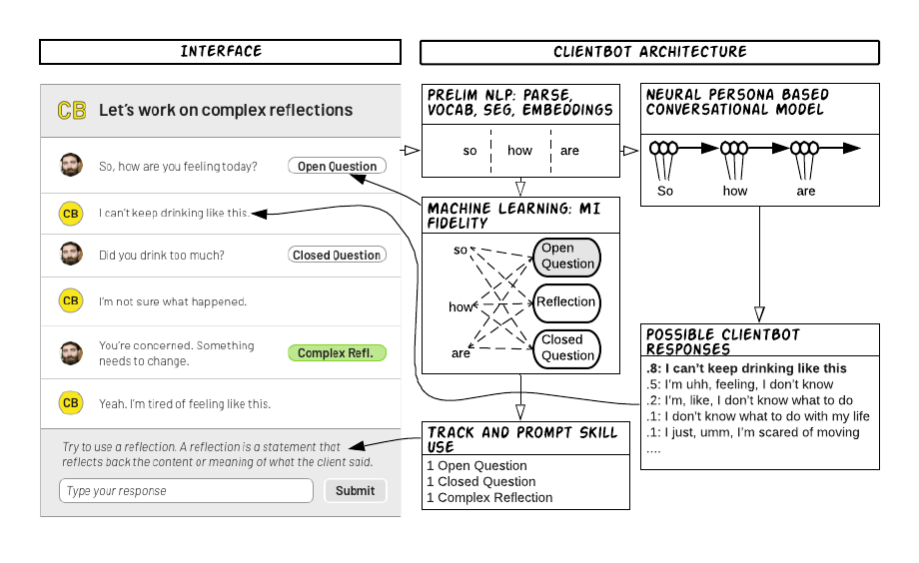
Client bot architecture.

To a trainee participant, the ClientBot platform appears like a standard chat interface, much like what a person might use if they were chatting on the Web with a friend or having a short message service (SMS) text message conversation on a mobile phone (see Figure 1). The key difference is that in this chat platform, the beginning therapist is interacting with a simulated patient, which responds to the trainee’s statements using neural network conversational models (described below). Although the trainee interacts with the simulated patient, ClientBot provides feedback on the individual’s chat responses, tailored to the skill they are currently practicing—either open questions or reflections. In the following sections, we describe the underlying models and development of the neural conversational model and machine learning–based feedback.

#### Long Short-Term Memory Cell

The simulated patient in ClientBot is a combination of 2 neural network systems with different strengths and limitations. Both rely on a long short-term memory (LSTM) cell, which is a variation on a traditional neural network sigmoidal unit that solves challenges in learning sequences with long-term dependencies. Similar types of models have been used for quite some time [33], but unfortunately, suffered from the “vanishing gradient problem” [34]. LSTM models solved many of these problems related to learning long sequences [35]. Here, we introduce the 2 LSTM recurrent neural networks underlying ClientBot and provide sample interactions.

The first model is a sequence to sequence model, similar to the model from the Vinyals study [28]. This class of models use an LSTM encoder network to read the input statement, and then have a separate, linked LSTM that decodes the statement into a response. Vinyals et al [28] adapted these models that were initially used for machine translation to be used in dialogue generation. The intuition is that dialogue can be thought of as a similar NLP problem to decoding a French sentence to an English sentence. This model is trained on a collection of English movie transcriptions (ie, open-subs dataset) [36] and 2354 psychotherapy transcripts published by Alexander Street Press, which is available for download with a library subscription [37]. The model was trained using adaptive gradient descent with a learning rate of .01 with gradient clipping of 5 [38], using a vocabulary of 15 thousand words and 1 layer of 3000 LSTM cells. This model achieved a perplexity of 9.06 on a random 5% of data that was not used during training. (Perplexity is a measure of how well the model predicted the next word in a sentence given the previous words, with lower values indicating better fit). When interacting with the model, it uses beam search decoding (with a beam size of 10). Each therapist statement was entered into the encoder, and the client statement was used as the output or criterion for the decoder model. The broad goal of choosing these 2 training sets was to provide the model conversational text, and in particular, the Alexander Street Press transcripts provided specificity of the content and nature of therapeutic conversation. Textbox 1 (Sequence to Sequence or seq2seq) shows example interactions from the model. These examples demonstrate that this model provides brief but plausible responses to questions, which are often indicative of “small talk.” However, this is also its limitation: It does poorly at providing longer responses, which would be typical from a client in a psychotherapy session. Another limitation is that it responds with the phrase “I don’t know” relatively frequently. Finally, because it is partly trained with a corpus of movie transcripts, it responds in ways that would be contextually rare in psychotherapy, such as “I love you.” As a result, after training the model, we undersampled responses with “I don’t know” and did not allow responses that contain “I love you.” These were the only manually developed adaptations for these models. Models were selected based on their accuracy predicting responses in a random 5% of the dataset using perplexity, a standard measurement of how well a language model fits the data. The seq2seq model achieved a perplexity of 9.06 (lower is better) on the test set of examples.

The second model underlying ClientBot is an LSTM model that is only trained on the Alexander Street Press psychotherapy dataset. This model inserted tags to indicate when a speaker was changing from a patient to a therapist and vice versa: (“<p>” and “<t>”). This model is a simpler variation of the seq2seq model. Instead of encoding and decoding, it is just predicting long sequences of words (50 word sequences for our training). The tags indicate the speaker changes and roles. This model used a learning rate of .05 and 1024 LSTM model hidden units in a single layer. On a 5% test set of the Alexander Street Press data, this model achieved a perplexity of 38.01 (note: perplexity is not comparable across different datasets and different vocabularies but rather used to compare models within a given dataset). The decoding for this model randomly sampled at each time step, instead of taking the best next word. The advantage of this strategy is that it increases the likelihood of seeing longer responses from the model, and it also makes the responses nondeterministic. Similar to the previous model, “I don’t know” is undersampled because of its frequency.

Textbox 2 (LSTM model) shows an example interaction with this second model simulating the role of the client. Compared with the first model, this model produces longer talk turns and is more likely to interject psychotherapy like topical matter.

For the ClientBot used in the training, we combined the 2 models by using the first model (sequence to sequence) for the first 5 responses with a trainee, after which the system switches to the second model (simple LSTM model). The reason for starting with the sequence to sequence model is that it generates conversation that often begins a session (eg, generic chitchat and general questions and responses). However, as the session progresses, these short responses are less useful than those generated in the second model, which is prone to discuss problems and symptoms at length.

**Example interaction with the Seq2Seq conversational model.**

1. Human: Hi
  Top 5 responses:
  - Hi.
  - How are you?
  - What are you doing here?
  - What are you doing?
  - How are you doing?
2. Human: Where are you from?
  Top 5 responses:
  - I don’t know
  - I’m from Ohio
  - From the north
  - I don’t know…
  - I don’t know…but I don’t know
3. Human: How old are you?
  Top 5 responses:
  - <number>
  - I’m <number>
  - I don’t know
  - <number>?
  - <number>, <number>
4. Human: Goodbye
  Top 5 responses:
  - Goodbye
  - Goodbye.
  - What are you doing?
  - Goodbye...
  - What are you doing here?

**Example interaction with the Simple long short-term memory conversational model.**

Model: I got a new prescription

Human: what prescription are you on

Model: well, I guess it was just like, I have some.

Human: you have some meds?

Model: yes.

Human: what else do you take?

Model: I take, uh, I take <number> pill, and then I take <number>. You know, and I think it was just one of those things.

Human: How do they make you feel?

Model: oh, my gosh.

Human: Good?

Model: yeah.

#### Machine Learning Prediction of Basic Counseling Skills

Another central feature of ClientBot is the ability to give immediate, performance–based feedback. In the present case, that feedback takes the shape of classifying each response from the trainee into basic counseling skills. Labeling of these skills was facilitated through a measure of MI, which includes specific behavior counts of several active listening skills. In this study, we focused on open versus closed questions and reflections. To do this, we utilized a discrete sentence feature machine-learning model that provided labels for each trainee statement [24]. The model was trained on a large set of transcripts from a psychotherapy dissemination study that focused on training counselors to do MI. Each transcript was rated using the MI skills code (MISC) [39] (see below in Measures section). The kappa scores for the machine-human agreement ranged between .39 and .79 and were close to human reliability on the codes of interest (open question, closed question, reflections) [24].

ClientBot was run on a server using Torch7 [40] with an Nvidia 980ti graphics processing unit (GPU), which drastically increased the speed of inference (an average example using 1 beam took 30 ms to run on the GPU. On a 4 core CPU, the same example took 500 ms). The time the system takes to identify each statement varies depending on the length of the utterance but was generally less than 1 second. Responses from the conversational agent took between 300 ms to 1 second, depending on which model was used. Participants generally commented that the system responded in a timely manner.

### Participants

For this proof-of-concept study, 151 nontherapists were recruited as participants to assess the effect of the interface on a population with no formal training in counseling. This population ensured that the participants are very unlikely to have been exposed to formal training in counseling skills previously. Participants for this study were recruited from Amazon Mechanical Turk (MTurk) [41]. We limited our sample to either “master workers,” who are workers that have a track record of high accuracy on the tasks on which they have worked in the past or workers with at least 10,000 approved jobs and a 95% overall approval rate. We also limited the sample to US residents who spoke US English and excluded participants under the age of 18 years. The amount that workers were paid depended on the demand for work at the time that they enrolled, which varied between US $3 and US $3.50 for each participant. Each potential participant was recruited to “practice your listening skills,” inviting interested people “...to chat with a simulated person for 20 min and practice their listening skills.” Participants then completed a short (10 question) survey when they were done.

### Procedure

Interested participants were directed to a page where they read the consent form. If they agreed to participate they were then randomized into 1 of 2 conditions. Both conditions were given a brief introduction to “listening skills,” focused on reflections and open questions including various examples. At the end, participants took a 3-question quiz to ensure that participants understood what skills they were supposed to be practicing. Users were allowed to go back and read this introduction after reading the questions.

**Figure 2:**
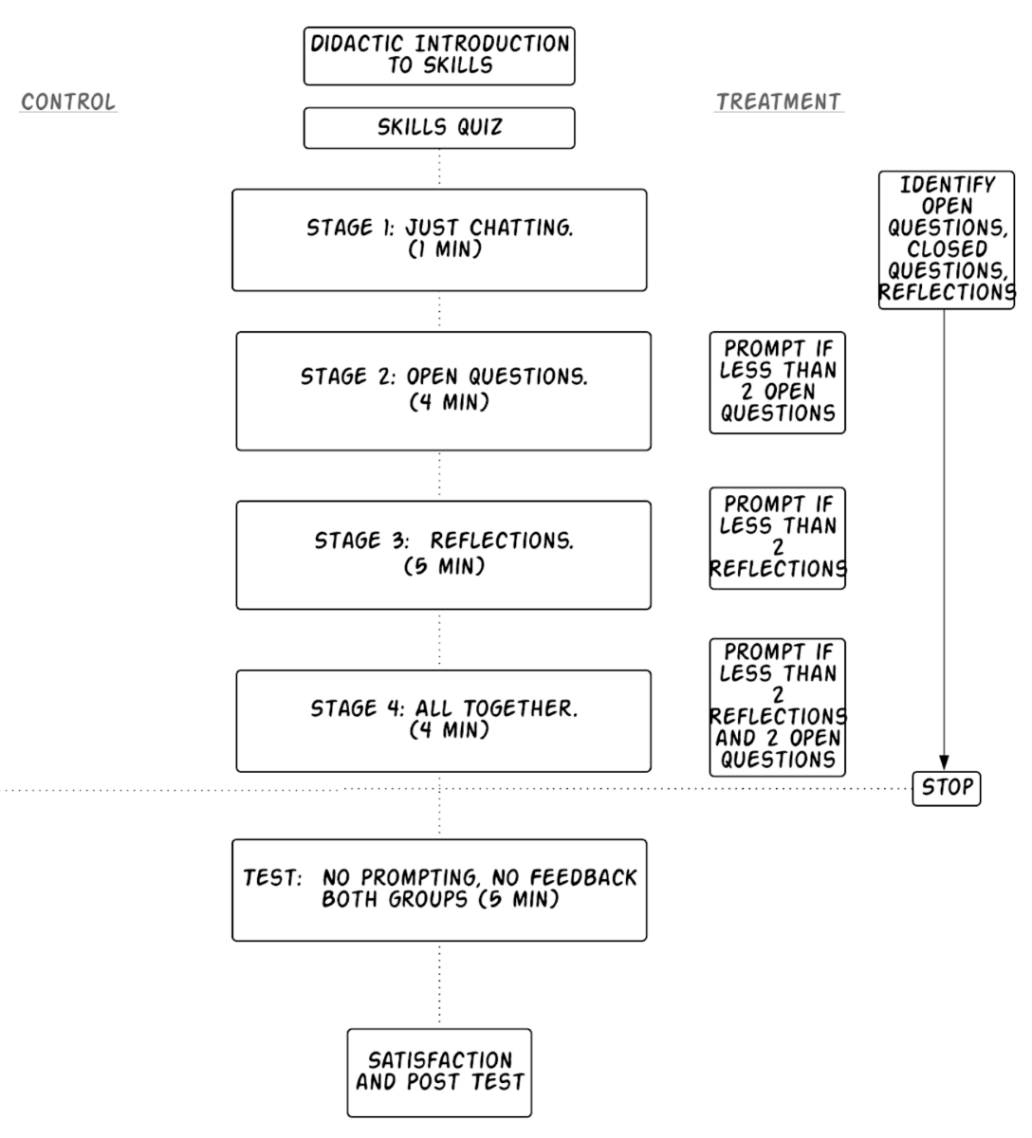
Client bot curriculum and design.

Both conditions included discrete phases focused on different skills, as shown in Figure 2. In the control condition, after reading the introduction, users began to interact with the simulated client. These users were prompted with skill-specific introductory prompts (eg, “now practice open questions,” and “now practice reflections”) but received no feedback on their interactions with the simulated client. Participants in the treatment condition read the same introductory statement, prompts, and training as the control condition (“now practice open questions”). If a user was not responding with, for example, enough reflections during the reflection training section, the system prompted them to practice more reflections and give examples similar to the introduction. All participants then had a 5-min test phase where all prompts and feedback are removed from the system (“For the last 5 minutes, show us your best listening skills”). After interacting with ClientBot, participants completed several questionnaires (see Measures).

Figure 2 shows the progression through the stages of the curriculum. Boxes in the middle of the figure represent components that both the treatment and control groups received. Items on the right side of the figure show components that only the treatment group received.

### Measures

#### Open Questions and Reflections

As noted above, ClientBot includes a machine-learning engine trained to identify categories of basic counseling skills assessed by a standard measure of MI fidelity [24]—the MISC [39]. We used models from the methods described in the study by Tanana et al [24]. These MISC identification models could identify open and closed questions and reflections on a test set with similar performance to human-human reliability (see [24] for full table of results). To track the success of training, the number of open question and reflection codes are tabulated and divided by the total number of utterances, yielding percentages of each type of statement. The primary outcomes during the training session itself were percentages of reflections and open questions.

#### Posttest Fixed Responses

The primary outcome to measure changes in MI desirable behaviors was the use of open questions and reflections during the interactive session with the simulated client. However, to guard against the possibility that the simulated person could create a self-reinforcing loop, exaggerating group differences, the users were also asked to respond to 5 standardized client responses during the posttest, using the skills they had learned (these prompts were exactly the same for all participants). After the completion of the curriculum, participants were asked to respond to 5 example client statements on a survey using the skills that they had learned. Their responses were coded using the automate fidelity system described above.

#### Satisfaction

We measured 2 types of satisfaction: (1) Satisfaction with the ClientBot system in general and (2) Satisfaction with the ClientBot simulated client. The first included questions such as “I thought the system gave me useful information” and “I would use the system again.” The second construct asked questions such as “The simulated person was interesting to talk to” and “I found the simulated person to be tedious to interact with.” All questions had the responses strongly agree, agree, disagree, and strongly disagree. Satisfaction scores were coded from 1 to 5 with 1 indicating strong dissatisfaction, 3 indicating a neutral response, and 5 indicating strong satisfaction.

### Data Analyses

Hypotheses 1 and 2 were tested by comparing the percentage of statements that were open questions and reflections between the treatment and control group (1) during the training phase and (2) during the test stage of the training (see Figure 2). The comparisons were made using a Wilcoxon rank test because the outcome was a transformation of a count variable.

## Results

### Performance

After consenting to the study, 22 users (12.1%) did not complete all phases of the interaction with the simulated client. This typically happened after only a few talk turns (median=9). There was no statistical difference in the rate of dropout between the treatment (15%) and the control group (10%; X^2^_1_ =0.51; *P*=.47). These users were excluded from all analyses.

A total of 151 participants completed the study (73 participants in the treatment group and 78 in the control group). The characteristics of the participants can be seen in Table 1. The sample was relatively balanced for males and females (53.6% male) and contained 25% non-white participants. The educational backgrounds were diverse as well, with 42% having a bachelor’s degree or higher.

**Table 1:** Demographics.

Identity status		Control, n (%)	Treatment, n (%)
**Race/ethnicity**			
	Native American	1 (1.3)	0 (0)
	Asian	3 (3.9)	6 (8.3)
	Black/African American	7 (9.1)	8 (11.1)
	Hispanic/Latino	6 (7.8)	3 (4.2)
	Multiracial	3 (3.9)	0 (0)
	White or not Hispanic	57 (74)	54 (75)
**Education**			
	High school or less	11 (14.3)	14 (19.4)
	Some college	32 (41.6)	25 (34.8)
	Bachelor’s degree or more	34 (44.2)	33 (45.8)
**Gender**			
	Female	38 (49.4)	31 (43.1)
	Male	39 (50.6)	41 (56.9)

At the outset of the study, after reading the initial introduction to open questions and reflections, participants answered 3 questions to test their understanding (Note that participants were able to go back and reread the introduction while answering these questions). The purpose of these questions was primarily a minimal validation check that participants were engaged in the task. A total of 96.6% of participants correctly answered the question about open questions, 95.3% the question about reflections, and 95.3% the question about the purpose of the study.

To verify that participants were not using a similar response repeatedly during their interactions with ClientBot, the percentage of unique utterances was estimated, with average of 98.5% unique utterances in treatment group and average of 97.3% unique utterances in control. This result indicates that very few participants could have repeated successful responses as a way of artificially inflating their performance or nominally completing the task without engaging in a meaningful way.

Performance of listening skills was assessed at 6 different time points during the study: before training began, during open question training, during reflection training, during combined reflection and open question training, after feedback was removed, and performance on fixed prompts on the posttest (see Tables 2 and 3). There were no differences in skill performance before training began on either open questions or reflections. During open question training, the treatment group used significantly more open questions than the control group (30.4% compared with 22.4%). During the reflection training, the treatment group used significantly more reflections than the control group (21.4% compared with 11.2%). During the combined training section, the treatment group used significantly more reflections than the control group (15.8% compared with 9.3%), but both groups used similar rates of open questions. After feedback was removed, the treatment group continued to use more reflections than the control group (14.1% compared with 8%), and both groups used similar rates of open questions.

Results in the posttraining assessment followed the same pattern as the responses with the simulated client. There were no significant differences in the use of open questions between the treatment and control group (W_149_=3040; *P*=.29). However, the treatment group used significantly more reflections than the control group (W_149_=1800; *P*<.01; *d*=0.58).

**Table 2:** Sample sizes for results.

Participant sample	Statistics, n	
	Control	Treatment
Before training	86	86
Open Question training	82	82
Reflection training	80	75
Training both	79	74
Test (feedback removed)	79	74
Fixed responses (posttest)	78	71

**Table 3:** Results of the assessment.

Task and Time Frame, (pre, training, post)	Open questions			Reflections			Reflection or open questions		
	Control	Treatment	*P* value	Control	Treatment	*P* value	Control	Treatment	*P* value
Before training	25.5	23.8	.97	8.6	8.6	.81	34.1	32.4	.77
Open Question training	22.4	30.4	<.001	6.3	5	.14	28.6	35.2	<.001
Reflection training	15.6	11	.003	11.2	21.4	<.001	26.8	32.4	.002
Training both	18.4	20.9	.07	9.3	15.8	<.001	27.7	36.8	<.001
Test (feedback removed)	16.7	18.3	.16	8	14.1	<.001	24.7	32.5	<.001
Fixed responses (posttest)	40	35.1	.29	18.2	34.6	<.001	58.2	60.7	.01

### Satisfaction

There were no significant differences between groups on overall satisfaction, satisfaction with the conversational agent, or satisfaction with the system in specific. Satisfaction was not significantly different from neutral (2.85; *t*_148_=–1.91; *P*=.06), with most of this effect attributed to dissatisfaction with the simulated person (2.39; *t*_148_=–6.28; *P*<.01) and a nearly neutral response to the system overall (3.02; *t*_148_=0.28; *P*=.77). There was no difference in system satisfaction by group (*t*_141_=0.021; *P*=.98), simulated person satisfaction by group (*t*_146_=0.552; *P*=.58), or overall satisfaction by group (*t*_144_=0.201; *P*=.84).

The majority of respondents said that system was not boring (70%) and that they thought the system gave useful information (75%). The participants were split on whether or not they would use the system again, with 46% reporting that they would. Only 35% of users thought that the simulated person was interesting to talk with, and a majority also thought that interacting with the simulated person was tedious (73%).

## Discussion

### Results Summary and Inferences

This study investigated a new methodology for teaching active listening skills to an untrained population using a computerized simulated patient, and automated feedback, that could all be delivered without experts supervising each individual directly. These initial results indicate that an untrained population can improve specific types of listening skills very quickly (in 20 min). The treatment group in this study had higher rates of reflections, and maintained their increased rate of reflections, even after the feedback and prompts went away. The control group showed an initial propensity to use open questions, even without feedback, but demonstrated a steady decay of open questions over time.

Surprisingly, there were no treatment effects for open questions. That is, only a brief introduction on open questions and some practice elicited use of open questions. These types of utterances can be produced by simply using a set of sentence stems (“How,” “Why,” “What”). As a result, an open question may be an easier skill to learn than a reflection, and less feedback is required. In contrast to open questions, reflections notably increased with feedback. A reflection involves listening to another person and responding with a summary or refrain of what that person has been trying to express. For example, if a client discussed concerns related to waking up with headaches and often missing work because of parties, a reflection might be to say, “so it sounds like you are worried that drinking is getting in the way of the things you would like to be doing in life.” For individuals who have never been exposed to MI or basic counseling skills training, reflections may be a less intuitive skill than an open question. In addition, during the survey following the training, some of the participants noted that they found it much more difficult to produce reflections than open questions.

This study primarily focused on the acquisition of 2 basic listening skills; however, there is some tentative evidence for the durability of the gains. After training, both groups took a satisfaction and demographic survey, and then were surprised with 5 more client statements that they were asked to respond to using the listening skills they had learned during the training. Although not a formal distraction task, the treatment group retained the skills from the training relative to the control; a promising result for later research into the durability of these gains.

Satisfaction is a secondary outcome compared with changes in the practice of skills but a potentially important factor for dissemination of a system such as the one tested in this study. Users in this study had a negative view of the simulated person and a neutral view of the system as a whole. It should be noted that there was no comparison with a more traditional curriculum that consisted purely of written material, and as a result, the view of satisfaction should be interpreted with caution. Users may have enjoyed this study’s experience more than the latter. However, the results suggest that efforts should be made to improve the user experience.

### Limitations and Future Directions

One important limitation of this study is that its participants were workers from Amazon MTurk. This is clearly a different population from students who might be starting a mental health training program (eg, social work, psychology, and psychiatry). However, this limitation presents some advantages; notably, these results should generalize to a wider population than just individuals who could be accepted to a counseling graduate program. One of the major limitations of typical psychology research is that it often relies heavily on undergraduate college populations, often limiting the generalization of this research. The MTurk population tends to more closely represent the US population and is much more diverse than the typical sample of undergraduate students [42]. However, further research should be conducted to verify that this type of approach can also benefit the population that does enter a graduate program in psychology.

This study primarily tested differences in acquisition of open questions and reflections and did not test differences in retention or transfer of learning. The Schmidt study [43] has pointed out that treatment differences in acquisition do not necessarily have an impact on retention and transfer. Future research should follow and test participants a week or more after treatment, possibly with multiple administrations of the treatment. Moreover, there should be an investigation into the effects of written training on spoken interactions. This study does not answer the question of how well practicing in a chat forum may transfer to an actual therapy setting. It is possible and even likely that many of the manipulations that have drastic improvements on acquisition may have much lower impacts on retention and transfer of learning.

There was a general sense among the participants of the study that the simulated patient was not a realistic substitute for another human. The computerized dialogue model could sometimes say distracting or irrelevant responses. It is important to note that these models were trained on a relatively small sample of dialogue compared with similar models published in the literature. For example, the Vinyals study [28] used 62 million training examples, whereas the corpus of psychotherapy transcripts used in this study only has 514,118 examples. Moreover, the dialogue in which these models trained was transcribed from actual spoken interactions, which tend to be filled with disfluencies and often trail off. Many of the original transcripts can be hard to understand for these reasons, so it is not surprising that the model trained on these transcripts can occasionally respond in a way that seems out of place. Despite the user’s dissatisfaction with the simulated person, the conversational agent did create thousands of novel utterances that the participant could use to practice their listening skills.

The bot performance might be improved by utilizing chat transcripts from Web–based therapy or crisis interventions via SMS text messages (eg, Crisis Text Line, Talk Space, and 7-Cups). Other sources of written text that might be relevant include the Reddit mental health–related forums; however, these function differently than traditional dialogue. This current conversational model is not able to track long-term topical dependencies in a dialogue but rather just attempts to create a likely response to the last talk turn. A more engaging and believable model will benefit from methods that can capture these long-term dependencies in a conversation. However, it may be possible that it is not entirely necessary for ClientBot to fully replicate the experience of talking to another human to provide a useful and satisfactory training experience. Ironically, there is evidence that as the bot begins to further approach a fully human-like presentation, it may become less satisfactory or odd (eg, the uncanny valley).

At a more conceptual level, ClientBot is a technology that is focused on supporting a human’s ability to communicate more effectively with other humans. Thus, its use raises fundamental questions about the relationship between humans and machines, or more specifically how humans function in these computer-supported learning environments. For example, it maybe that humans are more apt to trust feedback they receive from a computer (rather than a human) as they see it as more objective [44], even though machine learning–based ratings from a computer are prone to bias and error in a way commensurate with the data from which they are trained. [45]. Accordingly, it may be important to adapt future systems to help humans appropriate challenge the evaluations they receive [46]. Ultimately, the improvement of systems such as ClientBot will rely on ongoing “human in the loop” feedback [47], whereby users learn from the system and also provide feedback and insights that serve to make the platform more effective.

One interesting direction for this research is to possibly develop algorithms that can *produce* a sample reflection for a client statement. For example, in this study, when a trainee responds with an open question when they are supposed to be practicing reflections, the system may prompt them to “keep practicing reflections” and give a generic example of a reflection. In contrast, an ideal system may take the last client statement (“I just don’t know what to do, my work day never seems to end”) and give an example reflection (“you're feeling overwhelmed at your job”). It is not unreasonable to think that this type of model is plausible given the current state of NLP. NLP researchers have become excellent at question answering tasks [48], which is relatively similar to the problem of producing a reflection. Finally, this study examined both feedback on therapist talk turns as well as adaptive prompting. The treatment effects include both of these tools combined. In addition, both the control group and treatment group received prompting and a didactic explanation of the skills they were supposed to be practicing. Each of these components likely has an effect on the outcomes measured in the study. It would be beneficial to break down each of these skills into a separate component. This type of study would require a much larger pool of participants but would contribute useful knowledge about the impact of various training modalities.

### Conclusions

During the course of the last half century, fields such as aviation and medicine have used technology to augment and enhance the capabilities of humans. In contrast, psychotherapy training and practice generally look very similar to the way they did 50 years ago. Moreover, psychotherapy has the additional problem that there is no natural feedback loop providing practitioners with a means to improve over time [49]. This study tested a method for both providing feedback and training that has the possibility to scale beyond the time limits of a single expert trainer. The results show that at least for the population that participated in this study, that this methodology can improve performance of specific listening skills. This type of system presents a promising avenue to improve the scale on which feedback, adherence, and training can be delivered in the field of psychotherapy.

## Abbreviations

LSTM: long short-term memory
MI: Motivational Interviewing
MISC: motivational interviewing skills code
MTurk: Mechanical Turk
NLP: Natural Language Processing
SMS: short message service
